# Are the adverse effects of glitazones linked to induced testosterone deficiency?

**DOI:** 10.1186/1475-2840-7-30

**Published:** 2008-10-15

**Authors:** M Carruthers, TR Trinick, E Jankowska, AM Traish

**Affiliations:** 1 Centre for Men's Health, 20/20 Harley Street, London, UK; 2 Department of Chemical Pathology, The Ulster Hospital, Belfast, (TRT) UK; 3 Cardiology Department, Military Hospital, Wroclaw, Poland; 4 Institute of Anthropology, Polish Academy of Sciences, Wroclaw, Poland; 5 National Heart and Lung Institute, Imperial College, London, (EJ) UK; 6 Institute for Sexual Medicine, Boston University School of Medicine, Center for Advanced Biomedical Research, Boston, (AMT) USA

## Abstract

**Background:**

Adverse side-effects of the glitazones have been frequently reported in both clinical and animal studies, especially with rosiglitazone (RGZ) and pioglitazone (PGZ), including congestive heart failure, osteoporosis, weight gain, oedema and anaemia. These led to consideration of an evidence-based hypothesis which would explain these diverse effects, and further suggested novel approaches by which this hypothesis could be tested.

**Presentation of hypothesis:**

The literature on the clinical, metabolic and endocrine effects of glitazones in relation to the reported actions of testosterone in diabetes, metabolic syndrome, and cardiovascular disease is reviewed, and the following unifying hypothesis advanced: "*Glitazones induce androgen deficiency in patients with Type 2 Diabetes Mellitus resulting in pathophysiological changes in multiple tissues and organs which may explain their observed clinical adverse effects*." This also provides further evidence for the lipocentric concept of diabetes and its clinical implications.

**Testing of the hypothesis:**

Clinical studies to investigate the endocrine profiles, including measurements of TT, DHT, SHBG, FT and estradiol, together with LH and FSH, in both men and women with T2DM before and after RGZ and PGZ treatment in placebo controlled groups, are necessary to provide data to substantiate this hypothesis. Also, studies on T treatment in diabetic men would further establish if the adverse effects of glitazones could be reversed or ameliorated by androgen therapy. Basic sciences investigations on the inhibition of androgen biosynthesis by glitazones are also warranted.

**Implications of the hypothesis:**

Glitazones reduce androgen biosynthesis, increase their binding to SHBG, and attenuate androgen receptor activation, thus reducing the physiological actions of testosterone, causing relative and absolute androgen deficiency. This hypothesis explains the adverse effects of glitazones on the heart and other organs resulting from reversal of the action of androgens in directing the maturation of stem cells towards muscle, vascular endothelium, erythroid stem cells and osteoblasts, and away from adipocyte differentiation. The higher incidence of side-effects with RGZ than PGZ, may be explained by a detailed study of the mechanism by which glitazones down-regulate androgen biosynthesis and action, resulting in a state of androgen deficiency.

## Background

Recent clinical studies have raised serious concerns regarding the safety of glitazones, especially rosiglitazone (RGZ) and pioglitazone (PGZ) to regulate hyperglycemia in diabetic patients. A meta-analysis study [1] demonstrated use of RGZ was associated with a "*significant increase in the risk of myocardial infarction and with an increase in the risk from cardiovascular causes that had borderline significance*". These side effects were confirmed by other clinical studies[2] and meta-analyses[3], though some investigators, particularly those reporting the effects of PGZ treatment [4,5] showed reductions in cardiac deaths.

Because of the widespread use of glitazones, it is of considerable practical importance to understand the potential mechanisms underlying the differing effects of these two thiazolidines on clinical endpoints, in spite of their apparent similar effectiveness in reducing blood glucose, as well as their wide range of adverse side-effects, including weight gain, anaemia and osteoporosis. These links between the clinical, metabolic and endocrine effects of glitazones give rise to a unifying hypothesis based on reduction of testosterone biosynthesis and function

## Presentation of hypothesis

### A Unifying Hypothesis Linking the Adverse Effects of Glitazones to Induced Testosterone Deficiency

**Figure 1:**
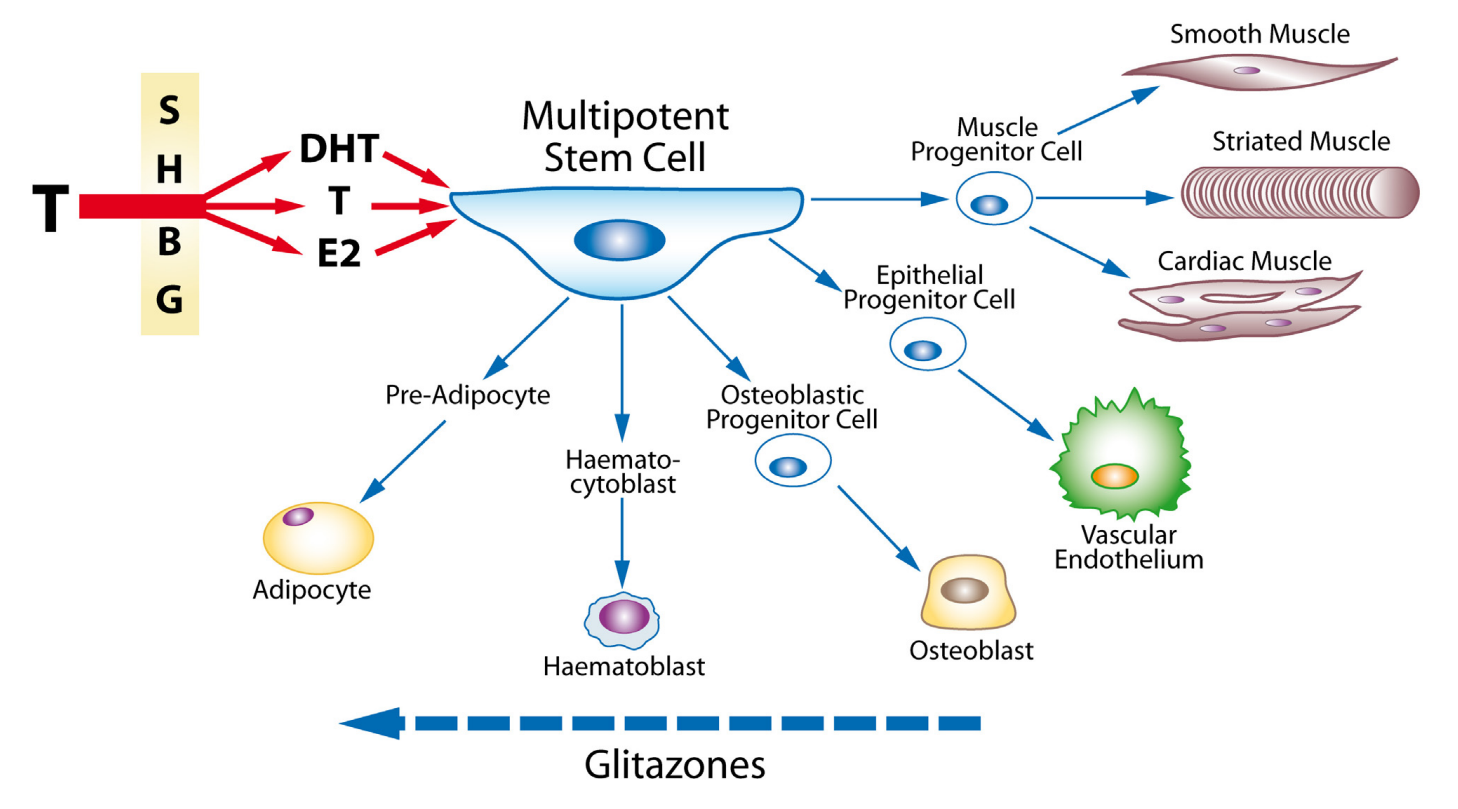
**Unifying hypothesis linking the adverse effects of glitazones to induced testosterone deficiency**. Testosterone, either directly or by conversion to dihydrotestosterone or oestradiol, all largely regulated by the effect of Sex Hormone Binding Globulin, acts on the Multipotent Stem Cell to promote differentiation to the progenitor cells for muscle, endothelium, bone, and red blood cells. By causing androgen deficiency, glitazones may reverse these effects and promote adipocyte production and action, with adverse clinical side-effects.

We advance the following unifying hypothesis: " *Glitazones induce androgen deficiency in patients with Type 2 Diabetes Mellitus resulting in pathophysiological changes in multiple tissues and organs which may explain their observed clinical adverse effects* ." (Figure 1).

It also provides further evidence for Ungar's theory of the 'Lipocentric Pathway to Hyperglycemia', and explains the toxic ectopic fat distribution in multiple organs, together with its clinical implications [6].

### Evidence Supporting this Hypothesis

#### A. Epidemiological Studies

There is increasingly considered that low T levels in men play an important role in the causation of T2DM, and are associated with reduced insulin sensitivity [7]. In men, circulating T is inversely related to classical cardiovascular disease (CVD) risk factors, including dyslipidaemia, hypertension, pro-thrombotic and pro-inflammatory states, insulin resistance, obesity, abdominal fat distribution, endothelial dysfunction, intima-media thickness of the carotid artery and thoracic aorta [8-12]. Men with coronary artery disease (CAD) confirmed by angiography have a markedly reduced level of circulating T as compared to those with normal coronary arteries [13-18].

#### B. Suppression Therapy

Management of prostate cancer via androgen-deprivation therapy with surgical or medical castration rapidly induces diabetes in susceptible individuals and is associated with cardiovascular events [19]. Androgen suppression therapy for prostate cancer has been linked to an increased incidence of coronary heart disease and risk factors for atherosclerosis [20].

#### C. Treatment in Men

**Figure 2:**
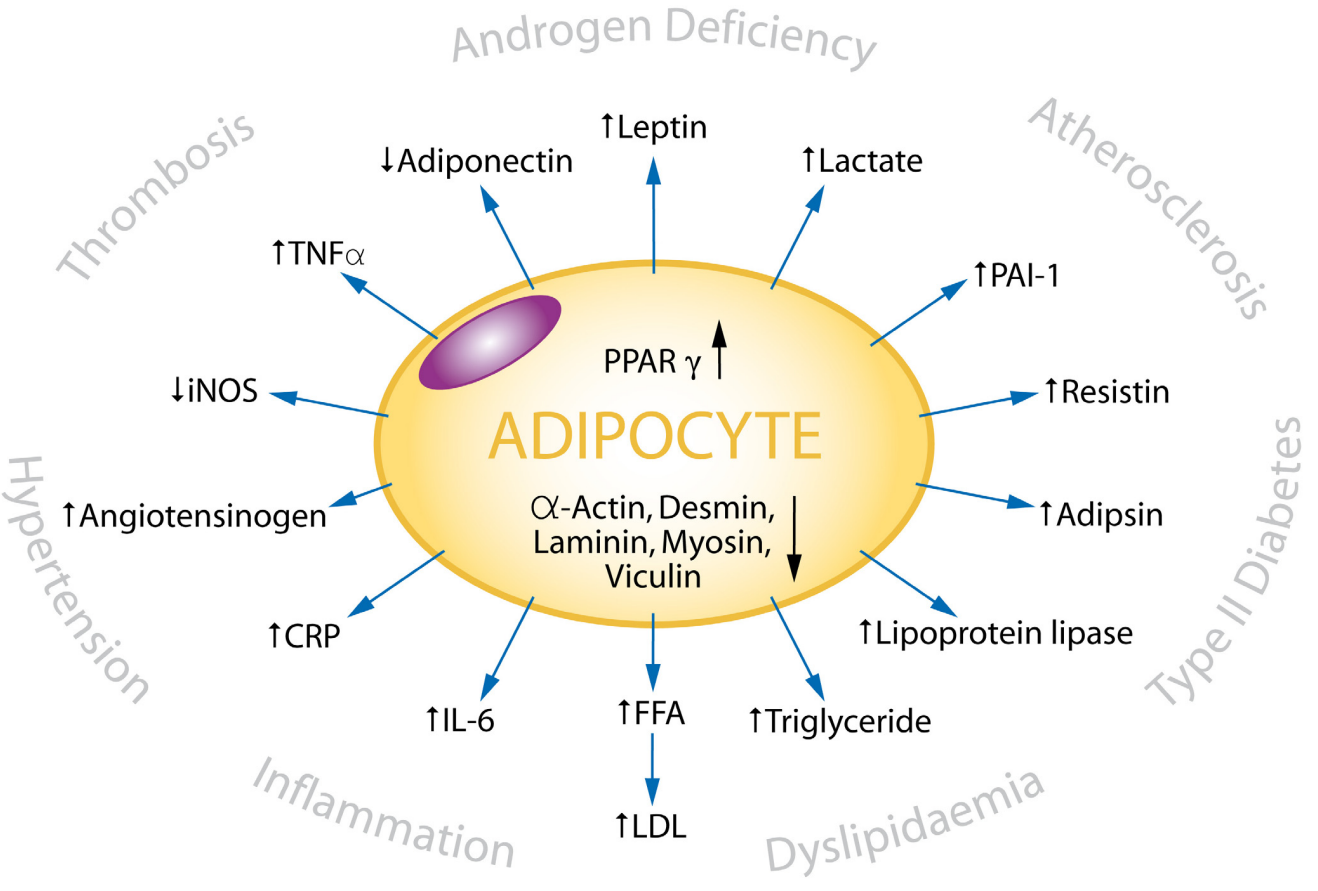
**The metabolic and clinical effects of adipocyte activity**. The adipocyte as the 'Axis of Evil' – PPARγ agonists such as the glitazones stimulate the adipocyte to produce adipocytokines and cause insulin resistance, dyslipidaemias, hypertension, and impaired immunological responses, which together can have the adverse clinical consequences shown.

Treatment of diabetic men with T has many beneficial effects, including increasing insulin sensitivity, correcting abnormalities in lipid metabolism, especially hypertriglyceridaemia, reducing visceral adiposity, decreasing leptin and adiponectin levels, reversing neuropathy, and improving erectile function. These effects are largely brought about by reducing the adverse metabolic effects of increased adipose tissue in organs throughout the body, but particularly in abdominal fat, reversing the actions of the adipocyte as the 'axis of evil' (Fig 2) [21,22] Beneficial clinical anti-ischaemic effects of T treatment in men with angina pectoris were reported as early as in the 1940s [23]. Acute T administration reduces exercise-induced myocardial ischaemia in men with CAD and low serum testosterone, also prolonging time to ST-segment depression [24-28].

#### D. Effects on Muscle and Adipocytes

Singh et al [29] suggested that androgens regulate the differentiation of multipotent stem cells into the myogenic lineage and inhibit adipogenesis. They also showed that T inhibited adipogenic differentiation of pre-adipocytes by activation of androgen receptor (AR)/beta-catenin interaction and translocation of androgen receptor/beta catenin complex to the nucleus, thus bypassing canonical Wnt signalling. These changes can affect all 3 forms of muscle:

##### Smooth muscle

Ultrastructural studies by Traish et al have documented that trabecular smooth muscle from castrated animals appears disorganized, with large number of cytoplasmic vacuoles and a decrease in myofilaments. Androgen deprivation in the animal model results in accumulation of adipocytes in penile tissues, particularly in the sub-tunical region [30]. T replacement restores normal cavernosal histological appearance. Recently, Kovanecz et al [31] have shown that treatment of obese diabetic Zucker fa/fa rats with PGZ produced globular fat-like cells in the corpus cavernosum especially at high doses. These observations together suggest a link between the function of anti-diabetic agents and interference with T action as shown in Fig. 1.

##### Striated Muscle

T increases lean body mass and decreases fat mass in young men, the magnitude of the changes being correlated with T concentrations. Especially in insulin resistant diabetes, impaired muscle strength and mass is likely to be associated with the reduction in myoglobin associated with low T levels.

##### Cardiac Muscle

Androgen receptors are present in the myocardium (cardiomyocytes) and vessel walls[32]. Their expression is modulated by catecholamines[33] and T itself, as shown by its depletion in hypertrophied and failing hearts, which is accompanied by deranged intracardiac steroid metabolism [34]. T deficiency is related to several changes within the myocardium, including impaired contractility of cardiomyocytes [34]. All these pathologies can be restored to normal on T supplementation.

#### E. Effects on Endothelial Progenitor Cells

T deficiency is associated with a low number of circulating progenitor cells and endothelial progenitor cells PCs in young men. T treatment induces an increase in these cells through a possible direct effect on the bone marrow [35].

#### F. Effects on Haemopoiesis

T treatment increases red blood cell production and hence haemoglobin and haematocrit either directly by promoting erythroid stem cell kinetics[36], or indirectly by its action on erythropoietin[37]. Patients with diabetes tend to be anaemic, especially the elderly, and their low T is correlated with their reduced haematocrit[38]. Treatment with RGZ and PGZ makes them more anaemic, which is probably related to lower T levels, not haemodilution [39].

#### G. Effects on Bone

Because osteoblasts and marrow adipocytes are derived from a common mesenchymal progenitor, increased adipogenesis may occur at the expense of osteoblasts, leading to bone loss. RGZ and PGZ usage were associated with more than doubling of fractures of the hip and wrist, increasing with the dose of either thiazolidine [40,41].

### Potential Mechanisms of Glitazone-Induced Androgen Deficiency

The actions of the glitazones on reductions in both TT and DHT have been shown in healthy men [42]. Troglitazone (TGZ) interferes with the activity of the P450 cytochrome oxidase (CPY) enzymes and was taken off the market in the USA because of its hepatotoxicity. It also increases sex hormone binding globulin (SHBG) which reduces FT[43]. As detailed in Fig. 1, T either directly, or by its conversion to DHT or estradiol, regulates the differentiation of multipotent stem cells into smooth, striated and cardiac muscle cells, osteoblastic/osteoclastic balance in bone, haemopoietic activity, and the formation of cytoskeletal components [30], while inhibiting the differentiation of progenitor cells into adipocytes.

The higher incidence of side-effects with RGZ than PGZ, may be further explained by a detailed study of the mechanism by which glitazones down-regulate androgen biosynthesis [44]. Both RGZ and PGZ changed the steroid profile of human adrenal NCI-H295R cells and inhibited the activities of P450c17 and 3betaHSDII, key enzymes of androgen biosynthesis. PGZ but not RGZ inhibited the expression of the CYP17 and HSD3B2 genes. Likewise, PGZ repressed basal and 8-bromo-cAMP-stimulated activities of CYP17 and HSD3B2 promoter reporters in NCI-H295R cells. However, PGZ did not change the activity of a cAMP-responsive luciferase reporter, indicating that it does not influence cAMP/protein kinase A/cAMP response element-binding protein pathway signalling.

There is also evidence that PGZ, to a greater extent than RGZ, increases the maturation of small adipocytes to larger ones, promoting a reduction in insulin resistance[45], decreasing lipogenesis in the liver, and increasing deposition of fat in the subcutaneous abdominal tissue, but not visceral fat. PGZ showed an additional beneficial effect on TG, HDL cholesterol and the levels of small dense LDL compared to RGZ [46].

## Testing of hypothesis

Clinical studies are needed to investigate the endocrine profiles, including measurements of TT, DHT, SHBG, FT and oestradiol, together with LH and FSH, in both men and women with T2DM before and after RGZ and PGZ treatment in double blind, placebo controlled groups. Also, further studies on T treatment in diabetic men would further establish if the adverse effects of glitazones could be ameliorated by androgen therapy. Basic sciences investigations on the inhibition of androgen biosynthesis by glitazones are also warranted.

## Implications of the hypothesis

The FDA reports that most PPAR agonists in development are non-thiazolidinediones, and though more than 50 Innovative New Drug (IND) applications have been filed for this group of drugs in the last seven years, most development programs have been terminated, all for safety reasons, and none have been approved.

This hypothesis explains the adverse effects of glitazones on the heart and other organs by reducing androgen action in directing stem cells differentiation into myocytes, vascular endothelium, erythroid stem cells and osteoblasts, and promoting adipocyte differentiation. The adverse clinical effects are directly linked to the metabolic actions of these drugs. The higher incidence of side-effects with RGZ compared with PGZ, may be explained by a detailed study of the mechanism by which glitazones down-regulate androgen biosynthesis and molecular mechanism of action, resulting in a state of androgen deficiency.

## Abbreviations

AR: androgen receptor; CHF: congestive heart failure; CVD: cardiovascular disease; DHT: dihydrotestosterone; FT: free testosterone; IL: interleukin; MI: myocardial infarction; PCs: progenitor cells; PGZ: pioglitazone; PPAR: Peroxisome Proliferator-Activated Receptor; RGZ: rosiglitazone; SHBG: sex hormone binding globulin; T: testosterone; TGZ: Troglitazone; TNF-α: tumour necrosis factor-alpha; T2DM: Type 2 diabetes mellitus; TT: total testosterone.

## Competing interests

The authors declare that they have no competing interests.

## Authors' contributions

MC conceived the unifying hypothesis. All authors contributed to the initial manuscript, and revisions were carried out by MC and AMT. All authors have read and approved the final manuscript.
